# New Classes of Mind Bomb-Interacting Proteins Identified from Yeast Two-Hybrid Screens

**DOI:** 10.1371/journal.pone.0093394

**Published:** 2014-04-08

**Authors:** Li-Chuan Tseng, Chengjin Zhang, Chun-Mei Cheng, Haoying Xu, Chia-Hao Hsu, Yun-Jin Jiang

**Affiliations:** 1 Institute of Molecular and Genomic Medicine, National Health Research Institutes, Zhunan Town, Miaoli County, Taiwan; 2 Laboratory of Developmental Signalling and Patterning, Institute of Molecular and Cell Biology, Singapore, Singapore; 3 Institute of Bioinformatics and Structural Biology, National Tsing Hua University, Hsinchu, Taiwan; 4 Biotechnology Center, National Chung Hsing University, Taichung, Taiwan; 5 Institute of Molecular and Cellular Biology, National Taiwan University, Taipei, Taiwan; Institute of Cellular and Organismic Biology, Taiwan

## Abstract

Notch signaling pathway defines an evolutionarily conserved mechanism in cell-fate determination in a broad spectrum of developmental processes through local cell interactions. *mind bomb* (*mib*) encodes an E3 ubiquitin ligase that is involved in Notch activation through Delta ubiquitylation and internalization. To further dissect the function of Mib, two yeast two-hybrid screens for zebrafish Mib/Mib2-binding proteins with different strategies have been performed. 81 putative interesting proteins were discovered and classified into six groups: ubiquitin proteasome pathway, cytoskeleton, trafficking, replication/transcription/translation factors, cell signaling and others. Confirmed by coimmunoprecipitation (Co-IP), Mib interacted with four tested proteins: ubiquitin specific protease 1 (Usp1), ubiquitin specific protease 9 (Usp9), tumor-necrosis-factor-receptor-associated factor (TRAF)-binding domain (Trabid)/zinc finger, RAN-binding domain containing 1 (Zranb1) and hypoxia-inducible factor 1, alpha subunit inhibitor (Hif1an)/factor inhibiting HIF 1 (Fih-1). Usp1, Usp9, Trabid and Fih-1 also bound to zebrafish Mib2, a Mib homolog with similar structural domains and functions. Both Mib and Mib2 can ubiquitylate Trabid and Fih-1, indicating a potential regulating role of Mib and Mib2 on Trabid and Fih-1 and, furthermore, the possible involvement of Notch signaling in hypoxia-regulated differentiation, tumorigenesis and NF-κB pathway. Finally, functions of confirmed Mib/Mib2-interacting proteins are collated, summarized and hypothesized, which depicts a regulating network beyond Notch signaling.

## Introduction

Notch signaling pathway is an evolutionarily conserved signal transduction cascade in flies, worms and vertebrates. It is a short-range cell communication. Through the Notch signaling pathway, signal-sending cells transfer a lateral inhibitory signal to the adjacent signal-receiving cells to control cell fate decision during development. It also plays roles in cell proliferation, cell death and self-renewal of adult stem cells [1]. The multiple functions of the Notch signaling pathway explain why improper Notch signaling causes human disorders such as Alagille syndrome, spondylocostal dysostosis and cancers [2]–[4].

Notch signaling is activated by the interaction of DSL ligands (Delta and Serrate for *Drosophila* and Lag-2 for *C. elegans*) on the surface of signal-sending cells with the Notch receptor on signal-receiving cells. Accompanying the interaction, ligands are internalized into cells that provide a pulling force on the Notch receptors leading to the exposure of the second cleavage site cut by ADAM metalloproteases. Then, the third cleavage of the Notch receptor by γ-secretase releases the Notch intracellular domain (NICD) into the nucleus. NICD forms a complex with CSL transcription factors (CBF1 for human; Suppressor of Hairless for *Drosophila* and Lag-1 for *C. elegans*) to regulate the expression of downstream genes [5]–[8].

**Figure 1:**
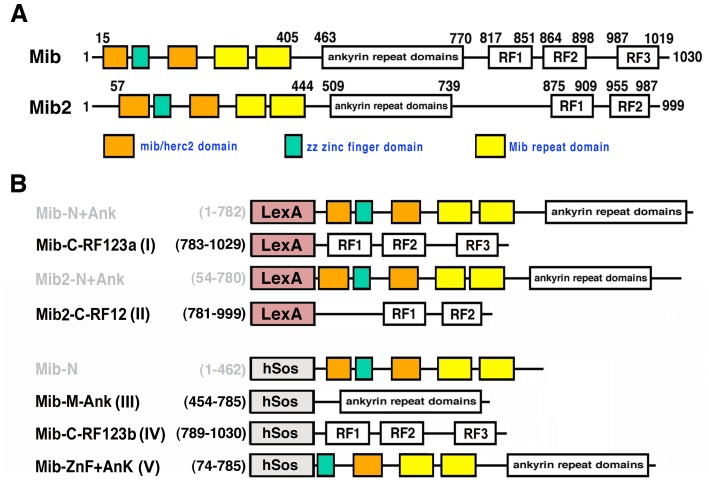
Structures of the bait proteins. Domain structures of Mib and Mib2 baits. (A) Schematic structures of Mib and Mib2 proteins. (B) Domain drawings of used baits. Autoactivated baits are in gray.

The endocytosis of DSL ligands is required for the activation of the Notch signaling pathway. The process is triggered by the ubiquitylation of ligands by Neuralized and Mind bomb (Mib). Mind bomb is an E3 ubiquitin ligase with two mib/herc2 domains, zz zinc finger domain, two mib repeats, eight ankyrin repeat domains (AND) and three RING finger domains ([9], Figure 1A). Mib in zebrafish can ubiquitylate and/or internalize ligands DeltaB, DeltaC, DeltaD, Jagged 1a, Jagged 1b and Jagged 2a [9]–[12]. This activity is essential for the Notch activation. Zebrafish *mib* mutants and *Mib*-deficient mice exhibit developmental defects in somites, neurons, pronephric duct, angiogenesis and heart due to a blockage in the Notch signaling pathway in early embryonic development [9], [12]–[14]. A paralog called Mind bomb 2 (Mib2) in zebrafish with similar protein structure to Mib but with only two RING finger domains (Figure 1A) also has the ability to ubiquitylate the ligand DeltaC [10]. Both Mib and Mib2 are able to auto-ubiquitylate themselves and form homodimer or heterodimer with each other. Overexpression of *mib2* in the zebrafish *mib* mutant can rescue the phenotypes, indicating a redundant role of Mib2 [15].

Ubiquitylation is a posttranslational modification with ubiquitin by the sequential action of ubiquitin-activating enzyme (E1), ubiquitin-conjugating enzyme (E2) and ubiquitin protein ligase (E3). Ubiquitin can be conjugated to substrates at many different sites and in many distinct topological configurations, which behaves as complex signals to regulate protein activity, localization and degradation [16]–[17]. This process is reversible and ubiquitin chain can be removed and edited by deubiquitylases (DUBs, also known as deubiquitinases, deubiquitylating enzymes or deubiquitinating enzymes). Therefore, the two-sided cooperation of ubiquitylation and deubiquitylation allows a precisely and immediately cellular response and control.

Studies have shown that both HIF activation and deregulated Notch signaling were linked to tumorigenesis and development [18]–[20]. Recent studies further emphasize the crosstalk between hypoxia and Notch signaling in cell differentiation [21]–[22]. Hypoxia blocks cell differentiation in a Notch-dependent manner through HIF-1α interaction with Notch intracellular domain and synergize to activate Notch downstream target genes [21].

The most well-known function of Mind bomb is its role in the Notch signaling pathway. However, it is not the only function of Mib. Mib has been reported to be involved in the apoptosis by interaction with death-associated protein kinase (DAPK) and cellular Fas-associated death domain (FADD)-like IL-1b converting enzyme (FLICE)-like inhibitory proteins (cFLIP) [23]–[24]. Mib also regulates the Wnt signaling pathway through the interaction with receptor-like tyrosine kinase (RYK) [25].

In this study, to further explore the function of Mib and Mib2, yeast two-hybrid screens were used to identify Mib/Mib2-interacting proteins. By this approach we isolated 81 putative Mib/Mib2-binding proteins and classified them into six groups: ubiquitin proteasome system, cytoskeleton, trafficking, replication/transcription/translation, cell signaling and others. The potential roles of Mib and Notch activity in these pathways require further investigation. Additional analysis was carried out on several interesting candidates. Hif1an/Fih-1 (hypoxia-inducible factor 1, alpha subunit inhibitor/factor inhibiting HIF 1) and Trabid/Zranb1 (tumor-necrosis-factor-receptor-associated factor-binding domain/zinc finger, RAN-binding domain containing 1) were shown to interact with and be ubiquitylated by Mib/Mib2 in COS7 cells. Usp1 and Usp9 can be co-immunoprecipitated by Mib/Mib2. These data suggest that Mib and Mib2 may be required for hypoxia-regulated differentiation, tumorigenesis and NF-κB pathway in addition to their prominent function in Notch signaling.

## Results

### 81 putative zebrafish Mib/Mib2-binding proteins were identified

To further investigate Mib function and identify putative Mib/Mib2-binding proteins, two yeast two-hybrid screens with different interaction strategies were performed using different regions of Mib and Mib2 as baits (Figure 1B) to screen the zebrafish whole embryo cDNA libraries. In the first screen, where the reconstitution of the functional transcription factor of LexA (DNA binding domain)-bait fusion protein and prey-Gal4 (activation domain) fusion protein activates the HIS3 reporter gene, 33 positive clones of 13 genes were identified by Mib-C-RF123a (783–1029 aa) from 60 million colonies, and 232 positive clones of 26 genes were identified by Mib2-C-RF12 (781–999 aa) from 52 million colonies (Table 1). Notably, there are much more positive clones identified by Mib2-C-RF12 (232 clones) than by Mib-C-RF123a (33 clones). With the bait Mib2-C-RF12, Mib was one of the positive clones, which is consistent with the finding that Mib2 is an interacting protein of Mib [10].

**Table 1:** Zebrafish Mib/Mib2-binding proteins isolated in the first yeast two-hybrid screen.

Prey Groups	Zebrafish Prey genes (GenBank accession number)	Human Homolog	Baits	PBS*	Preys Binding region (frequency)
Ubiquitin-Proteasome System	proteasome 26S subunit, non-ATPase, 1, (psmd1) (NM_201184)	PSMD1	I	D	114 - 320aa (2)
	ubiquitin specific peptidase 13 (isopeptidase T-3) (usp13) (NM_001098386)	USP13	II	C	438 - 889aa (3)
	ubiquitin B (ubb) (NM_001013272)	UBB	II	A	full length (31)
	mind bomb (mib) (NM_173286)	MIB1	II	D	507 - 876aa (1)
	ubiquitin A-52 residue ribosomal protein fusion product 1(uba52) (NM_001037113)	UBA52	II	A	full length (30)
	*ubiquitin specific protease 5 (usp5) (NM_214755)*	*USP5*	*II*	*A*	489 - stop codon (31)
	**ubiquitin specific protease 9 (usp9) (NM_001077449)**	**USP9**	**II**	**B**	1980 - 2363aa (3)
	ubiquitin C (ubc) (NM_001077804)	UBC	II	A	full length (45)
	ribosomal protein S27a (rps27a)/UBIQUITIN A-80-RESIDUE RIBOSOMAL PROTEIN FUSION PRODUCT (UBA80) (NM_200502)	RSP27A (UBA80)	II	A	full length (46)
Cytoskeleton	actinin alpha 2 (actn2) (NM_001037573)	ACTN2	I	D	528 - stop codon (1)
	actinin alpha 4 (actn4) (NM_199586)	ACTN4	I	A	660 - stop codon (7)
	septin 7a (sept7a) (NM_201161)	SEPT7	II	C	14 - 198aa (4)
Trafficking	SEC6-like 1; exocyst complex component 3 (exoc3) (NM_212715)	EXOC3 (SEC6)	I	B	full length (4)
	PREDICTED: Danio rerio similar to EPS15 protein (eps15) (XM_002663099)	EPS15	II	D	971 - stop codon (1)
	PREDICTED: Danio rerio epsin-2-like, transcript variant 1(epn2) (XM_681373)	EPN2	II	D	84 - 265aa (1)
Replication/Transcription/Translation	paired box gene 3 (pax3) (NM_131277)	PAX3 (HUP2)	I	D	192 - stop codon (2)
	paired box gene 7 (pax7a) (NM_131332)	PAX7 (HUP1)	I	D	112 - 397aa (1)
	paired box gene 7 (pax7c) (XM_689806)	PAX7 (HUP1)	I, II	D, D	141- stop codon (3)
	orthodenticle homolog 2 (otx2) (NM_131251)	OTX2	II	D	19 - 233aa (1)
	DEAD (Asp-Glu-Ala-Asp) box polypeptide 5 (ddx5) (NM_212612)	DDX5	I	A	47 - 273aa (6)
	PREDICTED: Danio rerio helicase-like transcription factor-like (hltf) (XM_687979)	HLTF (HIP116)	II	D	363 - 562aa (1)
Cell Signaling	TGF-beta activated kinase 1/MAP3K7 binding protein 3 (TAK1-binding protein 3) (tab3) (NM_001045105)	TAB3	II		338 - 611aa (1)
	polo-like kinase 4 (plk4) (NM_001118892)	PLK4	I	D	591 - 828aa (1)
	tyrosine 3-monooxygenase/tryptophan 5-monooxygenase activation protein, zeta polypeptide (ywhaz) (NM_212757)(14-3-3 zeta)	YWHAZ (14-3-3 zeta)	II	A	63 - 299aa (15)
	tyrosine 3-monooxygenase/tryptophan 5-monooxygenase activation protein, beta polypeptide b (ywhabb), mRNA (14-3-3 beta) (NM_213145)	YWHAB (14-3-3 beta)	II	D	76 - 263aa (1)
	GTP binding protein 4 (gtpbp4) (NM_199851)	GTPBP4 (CRFG)	II	D	43 - 502aa (1)
Others	4-aminobutyrate aminotransferase, gamma-aminobutyrate transaminase (abat) (NM_201498)	ABAT (GABAT)	I	D	156 - stop codon (1)
	plasma membrane calcium ATPase 4 (atp2b4) (EU559285)	ATP2B4 (PMCA4)	I	B	626 - 816aa (3)
	ankyrin repeat domain 52a (ankrd52a) (NM_001018154)	ANKRD52	II	D	230 - 510aa (1)
	insulin-degrading enzyme (ide) (NM_001089525)	IDE	II	B	252 - 813aa (7)
	family with sequence similarity 76, member B (fam76b) (NM_199932)	FAM76B	I	B	1 - 118aa (3)
	calcium binding protein 39-like (cab39l) (NM_001007327)	CAB39L	II	D	11 - 347aa (1)
	rapunzel (rpz) (NM_001145238)	unknown	II	D	47 - unknown (1)
	PREDICTED: ankyrin repeat domain-containing protein 13A-like (ankrd13a) (NM_001126387)	ANKRD13A	II	D	273 - stop codon (1)
	PREDICTED: Danio rerio similar to limb expression 1(lix1) (XM_681174)	LIX1	II	D	1 - 267aa (1)
	PREDICTED: Danio rerio zinc finger protein 569-like (LOC567317) (XM_690612)	unknown	II	D	11 - 235aa (1)
	PREDICTED: Danio rerio similar to Zinc finger protein 521 (LOC571623) (XM_695227)	unknown	II	D	96 - 221aa (1)
	similar to zinc finger like protein (LOC100330312) (XR_117615)	unknown	I	D	unknown (1)

**Notes.** Baits I: Mib-C-RF123a (783–1029 amino acid); II: Mib2-C-RF12 (781–999 amino acid).

*PBS (Predicted Biological Score): A = very high confidence in the interaction, B = high confidence in the interaction, C = good confidence in the interaction, D = moderate confidence in the interaction. The association of Mib/Mib2 and two of the preys (Usp5 and Usp9) were chosen for further investigation by co-immunoprecipitation (Co-IP). Preys that have or do not have physical interaction with Mib/Mib2 in Co-IP assay are in **bold** and *italic* fonts, respectively.

In the second screen with CytoTrap system, the bait and prey proteins are expressed and retained in the cytoplasm, where, unlike in the nucleus, they may undergo post-translational modifications. In addition, transcriptional activators and inhibitors may be used as baits to screen for protein-protein interactions. In this screen, we picked up a total of 95 positive clones that have in-frame cDNA sequence with the myristylation sequence, in which 52 clones of 28 genes by Mib-M-Ank (454–785 aa), 6 clones of 4 genes by Mib-C-RF123b (789–1030 aa) and 37 clones of 20 genes by Mib-ZnF+Ank (74–785 aa) (Table 2). Mib-M-Ank was screened for about 0.4 million colonies; Mib-C-RF123b and Mib-ZnF+Ank baits were individually screened for around 0.5 million colonies. Conspicuously, there are much more positive clones identified by Mib-M-Ank (52 clones) and Mib-ZnF+Ank (37 clones) than by Mib-C-RF123b (6 clones), which is consistent with the previous report that N-terminus and middle ankyrin repeats, but not C-terminal RF123, are the major binding domains of Mib protein to itself or other interacting proteins [10]. The interacting candidates through Mib-M-Ank and Mib-ZnF+Ank include both unique (19 genes and 11 genes, respectively) and common clones (9 genes) (Table 2).

**Table 2:** Zebrafish Mib-binding proteins isolated in the second yeast two-hybrid screen.

Prey Groups	Zebrafish Prey genes (GenBank accession number)	Human Homolog	Baits	Preys Binding region (frequency)
Ubiquitin Proteasome System	**ubiquitin specific protease 1 (usp1) (NM_199579)**	**USP1 (UBP)**	**V**	349 - stop codon (1)
	proteasome subunit, alpha type, 5 (psma5) (NM_205708)	PSMA5	V	from start codon (1)
	proteasome 26s subunit, non-ATPase, 4 (psmd4) (NM_001002112)	PSMD4 (Rpn10)	III, V	from 80aa (3)
	**tumor-necrosis-factor-receptor-associated factor-binding domain (trabid)/zinc finger, RAN-binding domain containing 1 (zranb1) (NM_001077768)**	**ZRANB1 (TRABID)**	**III**	from 295 - stop codon (1)
Cytoskeleton	myosin light chain, phosphorylatable, fast skeletal muscle a (mylpfa) (NM_131188)	MYLPF	III, V	full length (21)
	atrial myosin light chain (AF434191)	unknown	III	full length (1)
	actinin alpha 3 (actn3) (NM_131522)	ACTN3	V	680- stop codon (1)
	cofilin 1 (non-muscle) (cfl1) (NM_213639)	CFL1	V	9 - stop codon (3)
	type I cytokeratin, enveloping layer, like (cyt1l) (NM_001082882)	unknown	III, V	from 157aa (3)
Trafficking	exocyst complex component 1 (exoc1) (NM_199597)	EXOC1 (SEC3)	III	from 305aa (1)
	vesicle docking protein p115 (vdp) (NM_200155)	USO1 (VDP)	III	from 600aa (1)
	translocase of outer mitochondrial membrane 20 homolog b (yeast) (tomm20b) (NM_001002698)	TOMM20 (MOM19)	III, V	from15aa (2)
Replication/Transcription/Translation	Danio rerio c20orf14 homolog (H. sapiens), mRNA (c20orf14) (BC066556)	PRPF6	V	from 430aa (2)
	cleavage and polyadenylation specific factor 3 (cpsf3) (BC085402)	CPSF3	III	from start codon (2)
	replication protein A (rpa) (NM_131711)	RPA2 (HSSB)	V	from start codon (3)
	splicing factor 3a, subunit 3 (sf3a3) (BC096781)	SF3A3 (PRP9)	V	from 302aa (2)
	poly A binding protein, cytoplasmic 1 a (pabpc1a) (NM_001031676)	PABPC1 (PABP)	V	from 449aa (1)
	v-maf musculoaponeurotic fibrosarcoma (avian) oncogene homolog (maf) (BC065941)	MAF	III, V	from 136aa (3)
	*atonal homolog-1 (zath-1) (AF024536)*	*ATOH1 (ATH1)*	*V*	from 38aa (1)
	ribosomal protein L27 (rpl27) (NM_199724)	RPL27	III	from start codon (1)
	ribosomal protein, large, P0 (rplp0) (NM_131580)	RPLP0	III	from 157aa (2)
	ribosomal protein L17 homolog (rpl17) (NM_212760)	RPL17	IV	full length (1)
Cell Signaling	**hypoxia-inducible factor 1, alpha subunit inhibitor (hif1an)/factor inhibiting HIF 1 (fih-1) (NM_201496)**	**HIF1AN (FIH1)**	**III**	full length (1)
	tyrosine 3-monooxygenase/tryptophan 5-monooxygenase activation protein, theta polypeptide a (ywhaqa) (14-3-3 theta) (BC066409)	YWHAQ (14-3-3 theta)	III	57 - stop codon (1)
	low density lipoprotein receptor-related protein associated protein 1 (lrpap1) (NM_201306)	LRPAP1	V	from 43aa (1)
	B-cell receptor-associated protein 31 (bcap31) (NM_200092)	BCAP31 (BAP31)	III	from start codon (1)
	poly ADP-ribose polymerase 12-like, (LOC564886) (XM_688209)	unknown	IV	938 - stop codon (3)
	progesterone receptor membrane component 1 (pgrmc1) (NM_001007392)	PGRMC1 (MPR)	III	full length (1)
	creatine kinase, muscle (ckm) (NM_130932)	CKM (CKMM)	III	from 55aa (1)
	brain-subtype creatine kinase (ckbb)(AY055849)	CKB (BCK)	III	from 169aa (1)
	casein kinase 2 beta (ck2b) (NM_131187)	CSNK2B	V	full length (1)
Others	YY1 associated factor 2 (yaf2) (NM_001045268)	YAF2	III	116 - stop codon (1)
	apolipoprotein A-I (apoa) (NM_131128)	APOA1	IV	from start codon (1)
	apolipoprotein Eb (apoeb) (BC065592)	APOE	III	from start codon (1)
	nascent polypeptide-associated complex alpha polypeptide (naca) (BC122432)	NACA	III, V	from 101aa (7)
	immunoglobulin binding protein (AY648749)	HSPA5	III, V	483 - stop codon (4)
	glyceraldehyde-3-phosphate dehydrogenase, transcript variant 1 (gapdh) (XM_679205)	GAPDH	III	from 59aa (1)
	GDP dissociation inhibitor 2 (gdi2) (AY391428)	GDI2	III, V	from 279aa (3)
	*heat shock cognate (hsc70) (L77146)*	*HSPA8 (HSC70)*	*III*	420 - stop codon (2)
	heat shock 70 kDa protein 5 (glucose-regulated protein) (hspa5) (BC063946)	HSPA5 (GRP78)	III, V	from 478aa (4)
	heat shock protein 90 kDa alpha (cytosolic), class B member 1 (hsp90ab1) (NM_131310)	HSP90AB1 (HSP84)	III	from start codon (1)
	heat shock protein 9 (hspa9) (NM_201326)	HSPA9	III	from 513aa (1)
	PREDICTED: Danio rerio wu:fc5af04, transcript variant 1(wu:fc51f04) (XM_680524)	unknown	IV	431 - stop codon (1)

**Notes.** Baits III: Mib-M-Ank (454–785 amino acid); IV: Mib-C-RF123b (789–1030 amino acid); V: Mib-ZnF+Ank (74–785 amino acid). The association of Mib and five of the preys (Usp1, Trabid, Zath-1, Fih-1 and Hsc70) were chosen for further investigation by co-immunoprecipitation (Co-IP). Preys that have and do not have physical interaction with Mib in Co-IP assay are in **bold** and *italic* fonts, respectively.

In these two screens, the baits Mib-N (1–462 aa), Mib-N+Ank (1–782 aa) and Mib2-N+Ank (54–780 aa) showed autoactivation and were not used for screens to avoid false positive clones. Interestingly, all of these autoactivated baits contains two mib/herc2 domains, whose configuration and/or conformation may have unexpected higher affinity with or non-specific binding to the reporter constructs or non-binding activation of Ras signal. In the comparison of these two screens, many positive clones are functionally related and can be classified into six groups: ubiquitin proteasome system, cytoskeleton, trafficking, replication/transcription/translation, cell signaling and others (Tables 1 and 2). Although no identical gene was found from both screens, interestingly, the isoforms of tyrosine 3-monooxygenase/tryptophan 5-monooxygenase activation protein (14-3-3): 14-3-3 beta (β), 14-3-3 zeta (ζ) and 14-3-3 theta (θ) appeared in these two screens. 14-3-3 zeta was picked up for fifteen times by the bait Mib2-C-RF12 in the first screen. In addition, the family of actinin and paired box genes were also identified with different baits. In these two yeast two-hybrid screens, totally, 81 putative interacting proteins were isolated, implying that Mib and Mib2 may be involved in many cellular events that are intriguing to be investigated further.

Some genes are frequently picked-up. Those that have more than 5 clones identified include *ribosomal protein S27a* (*rps27a*)/*ubiquitin A-80-residue ribosomal protein fusion product* (*UBA80*) (46), *ubiquitin C* (*ubc*) (45), *ubiquitin B* (*ubb*) (31), *ubiquitin specific protease 5* (*usp5*) (31), *ubiquitin A-52 residue ribosomal protein fusion product* (*uba52*) (30), *myosin*, *light chain*, *phosphorylatable*, *fast skeletal muscle a* (*mylpfa*) (21), *tyrosine 3-monooxygenase/tryptophan 5-monooxygenase activation protein*, *zeta polypeptide b* (*ywhaz*) (*14-3-3 zeta*) (15), *actinin alpha 4* (*actn4*) (7), *insulin-degrading enzyme* (*ide*) (7), *nascent polypeptide-associated complex alpha polypeptide* (*naca*) (7), *DEAD (Asp-Glu-Ala-Asp) box polypeptide 5* (*ddx5*) (6). They belong to either proteasome/ubiquitin system or structural housekeeping genes, except *14-3-3 zeta*, *ide*, *naca* and *ddx5*. The higher number of clones may reflect the abundance of these genes.

### Mib and Mib2 interact with deubiquitylases Usp1, Usp9 and Trabid

In our screens, the ubiquitin proteins: ubiquitin B, ubiquitin C, ubiquitin A-52 and ubiquitin A80 appeared with a high frequency. Proteasome subunits including proteasome 26S subunit 1 (psmd1), proteasome subunit 5 (psma5) and proteasome 26S subunit 4 (psmd4) were also identified from both screens, reflecting a role of protein degradation and the E3 ligase activity of Mib and Mib2. Other intriguing interacting proteins were deubiquitylases (DUBs): ubiquitin specific protease 1 (Usp1), ubiquitin specific protease 5 (Usp5), ubiquitin specific protease 9 (Usp9) and tumor-necrosis-factor-receptor-associated factor-binding domain (Trabid) in the ubiquitin proteasome system group (Tables 1 and 2).

**Figure 2:**
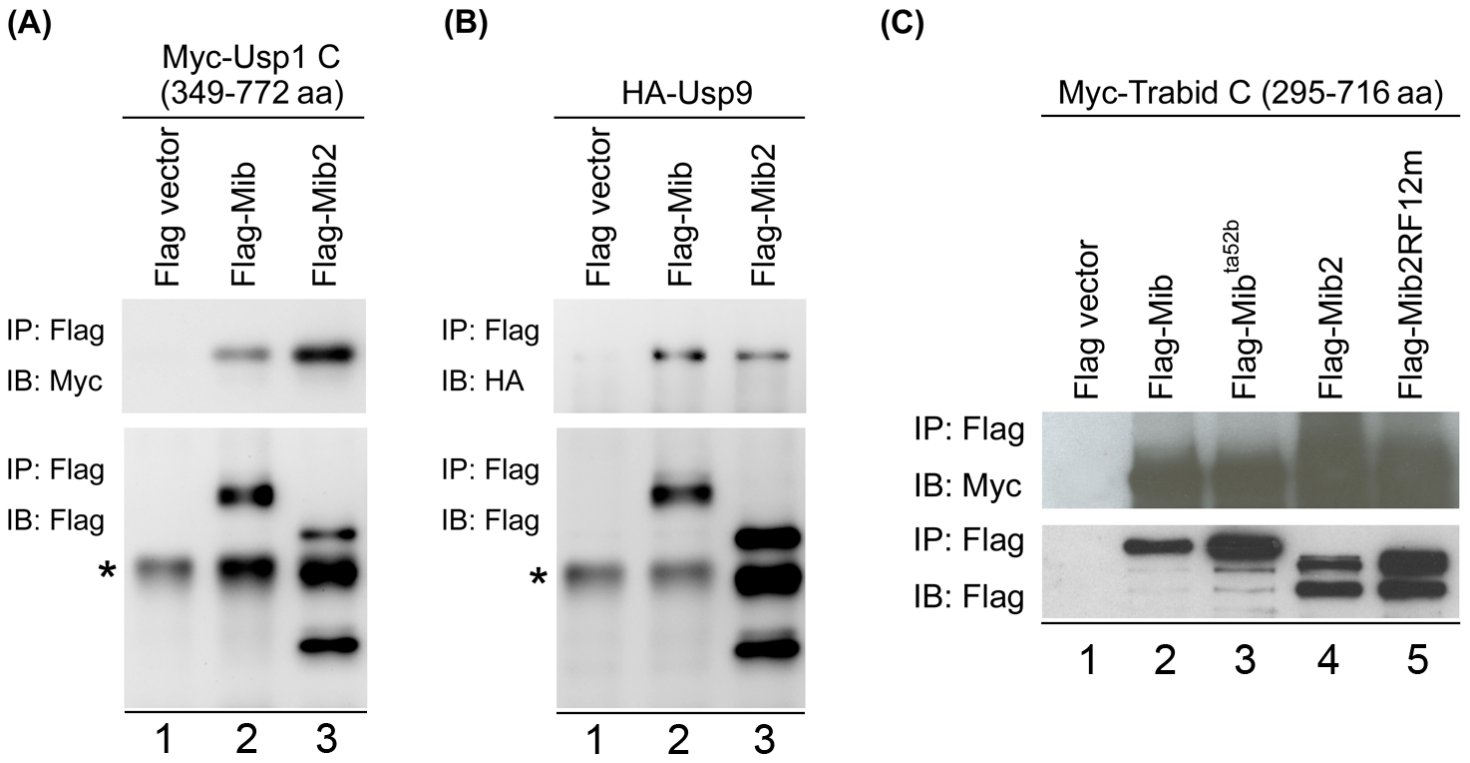
Interaction of Mib and Mib2 with Usp1, Usp9 and Trabid. Immunoprecipitation of (A) Usp1 C (349–772 aa), (B) Usp9 (full length) and (C) Trabid C (295–716 aa) by Mib and Mib2. COS7 cells were co-transfected with the expressing plasmids for HA- or Myc-tagged DUBs and FLAG-tagged Mib/Mib2. Immunoprecipitation (IP) were performed with anti-FLAG M2-Agarose and detected by Western blotting (IB) with anti-FLAG, anti-Myc or anti-HA antibody. Star indicates non-specific signals.

Ubiquitylation is a reversible reaction that ubiquitin can be removed by deubiquitylases. DSL ligands can be ubiquitylated by Neuralized and Mib. However, deubiquitylation of ligands is fully unknown. Usp1, Usp5, Usp9 and Trabid picked out from our screens might be potential candidates to regulate the Notch signaling pathway. To further verify the physical interactions of Mib with these deubiquitylases, co-immunoprecipitation was performed in COS7 cells. Cells were coexpressed with different FLAG-tagged *mib* or -*mib2* and Myc- or HA- tagged *DUBs*. The whole cell lysate was immunoprecipitated (IP) by anti-FLAG antibody and then subjected to Western blotting (IB) by anti-Myc antibody or anti-HA antibody to detect the interacting proteins. The results indicated that Usp1 C (from 349 to 772 amino acids), Usp9 and Trabid C (from 295 to 716 amino acids) were co-immunoprecipitated with Mib (Figure 2A, lane 2; Figure 2B, lane 2; Figure 2C, lane 2), but Usp5 was not (data not shown). Because Mib and Mib2 are E3 ubiquitin ligase in Notch activation through Delta ubiquitylation and endocytosis [9]–[10], [14], [26]–[28] and zebrafish Mib and Mib2 have both common and specific Delta substrate [10], the interaction of Mib2 and these DUBs was also examined by immunoprecipitation. The results showed that Usp1 C, Usp9 and Trabid C were also co-immunoprecipitated with Mib2 (Figure 2A, lane 3; Figure 2B, lane 3; Figure 2C, lane 4), but Usp5 was not (data not shown). Mib^ta52b^ and Mib2RF12m (E3 ligase-inactivated form, point mutation in the C-terminal-most RF [10] also bound to Trabid C (Figure 2C, lanes 3 and 5), reflecting that E3 enzymatic activity is not required for binding.

### Mib and Mib2 interact with Fih-1 that is involved in the hypoxia signaling pathway

**Figure 3:**
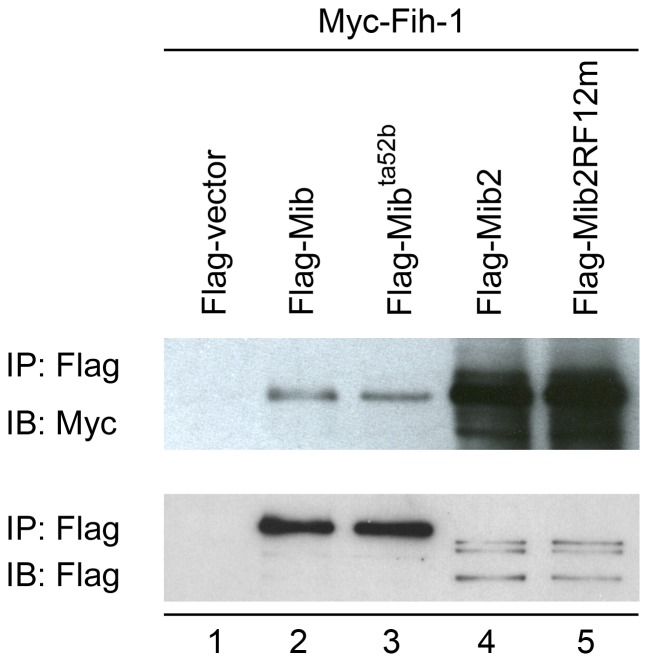
Interaction of Mib and Mib2 with Fih-1. IP of Fih-1 by various Mibs and Mib2s. COS7 cells were co-transfected with pCS2-FLAG tag and pCS2-MT tag constructs. The cell lysate was incubated with anti-FLAG M2-Agarose and then subjected to IB with anti-Myc (top panel) or anti-FLAG antibody (bottom panel).

In addition to DUBs, interacting candidates: atonal homolog-1 (Zath-1), heat shock cognate (Hsc70) and factor inhibiting HIF-1 (Fih-1) were investigated to verify their binding activities with Mib and Mib2 for the reasons that they have been shown to interact with Notch signaling genetically and/or biochemically [21], [29]–[31]. Results of immunoprecipitation done in COS7 cells indicated that Fih-1 is able to interact with Mib and Mib2 physically (Figure 3, lanes 2 and 4), but neither Zath-1 nor Hsc70 was pulled down by Mib or Mib2 (data not shown). Similar to Trabid, Mib^ta52b^ and Mib2RF12m also bound to Fih-1 (Figure 3, lanes 3 and 5).

### Trabid and Fih-1 are substrates of Mib and Mib2

**Figure 4:**
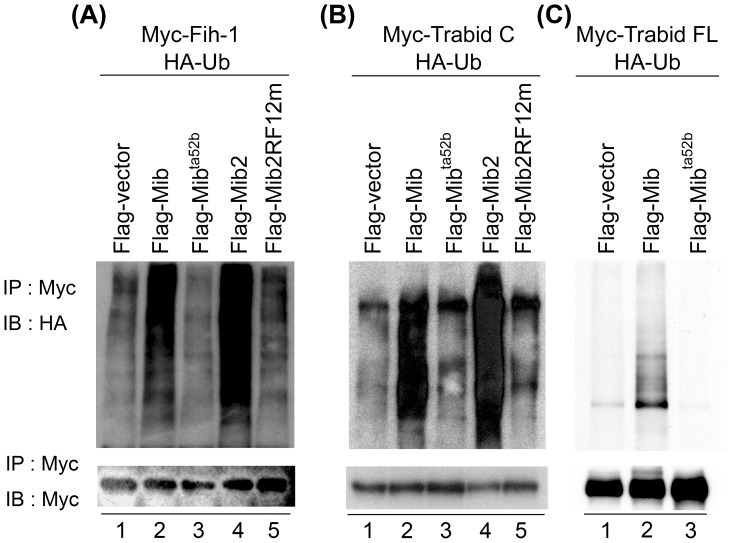
Both Mib and Mib2 promote Fih-1and Trabid ubiquitylation. (A) Ubiquitylation of Fih-1, (B) Trabid C (295–716 aa) and (C) Trabid FL (full-length) by Mib or Mib2. COS7 cells were co-transfected with pcDNA3.1-HA-ubiquitin, pCS2-FLAG tag and pCS2-MT tag constructs. The cell lysate was incubated with anti-Myc antibody and then subjected to IB with anti-HA (top panel) or anti-Myc antibody (bottom panel).

Zebrafish Mib and Mib2 are E3 ubiquitin ligase and can mediate ubiquitylation of their substrates in a most C-terminal RF-dependent manner [10]. Since both Mib and Mib2 can bind to Fih-1 and Trabid, we next asked whether Mib and Mib2 could promote the most C-terminal RF-dependent substrate ubiquitylation. COS7 cells were cotransfected with pcDNA3.1-HA-*ubiquitin*, different pCS2-FLAG-*mib* or -*mib2* and pCS2-MT-*fih-1* or -*trabid C* (295–716 aa). The whole cell lysate was immunoprecipitated with anti-Myc antibody and then subjected to immunoblotting with anti-Myc antibody as a control or anti-HA to detect the ubiquitylated proteins. Both Mib and Mib2 facilitated Fih-1 and Trabid C ubiquitylation (Figure 4A and 4B, lanes 2, 4), while the E3 ligase-inactivated Mib^ta52b^ or Mib2RF12m failed to do so (Figure 4A and 4B, lanes 3, 5). Trabid FL (full length) was also ubiquitylated by Mib (Figure 4C). These data imply that in addition to DeltaC, zebrafish Mib and Mib2 have two novel common substrates: Fih-1 and Trabid.

## Discussion

Yeast two-hybrid screen is a feasible approach to discover binding/interacting proteins and unexplored functions of Mib/Mib2. 81 putative interacting proteins were identified in our yeast two-hybrid screens. Most of them are novel. The interaction of Usp1, Usp9, Trabid, Fih-1 and Mib/Mib2 were confirmed by immunoprecipitation. Notably, the interacting proteins identified from Mib were also co-immunoprecipitated with Mib2, and vice versa (Figures 2 and 3). We also demonstrated that Trabid and Fih-1 are substrates of Mib and Mib2. Our screens showed that Mib and Mib2 are likely to interact with proteins involved in different biological processes, including ubiquitin proteasome system, cytoskeleton, trafficking, replication/transcription/translation, cell signaling and others. It reveals the roles of Mib and Mib2 are versatile and not just limited to the Notch signaling pathway.

In these two screens, putative interacting proteins of Mib/Mib2 are classified according to their biological functions, but there is no identical clone detected from both screens. The reason is likely due to their different screen strategies. While in the first screen, the interaction of bait and prey occurred in the nucleus by reconstitution of binding and activating domains of transcription factors; in the second, it took place close to plasma membrane by restoration of a functional Ras signaling pathway. In addition, the baits for the first screen were Mib-C-RF123a and Mib2-C-RF12, which are not major binding domains [10]; and much more interacting proteins were identified by baits Mib-M-Ank and Mib-ZnF+Ank than Mib-C-RF123b in the second screen. Furthermore, the screening cDNA libraries are different. Last but not least, different degree of saturation in screen is another possibility that cannot be ruled out. All of these parameters can affect the outcomes of these two yeast two-hybrid screens.

### E3 ligases and deubiquitylases (DUBs)

Ubiquitin and proteasome subunits are binding proteins of Mib and Mib2 identified in our screens. It reflects a role of Mib/Mib2 in proteasome-dependent degradation of their substrates such as DAPK and RYK [23], [25]. In this study, we demonstrated that Usp1, Usp9 and Trabid are interacting proteins of Mib and Mib2 by yeast two-hybrid and immunoprecipitation. DUB/E3 interaction has been reported that it can fine-tune protein degradation on their common substrates and/or stabilize the autoubiquitylated E3 ligase. For example, USP7 (HAUSP) interacts with MDM2 to regulate the turnover of p53 together, and it is also able to increase the stability of MDM2 [32]. USP9X (FAM) deubiquitylates the autoubiquitylated E3 ligases Itch and SMURF1 and, thereby, increases their stability [33]–[34]. Mib/Mib2 with autoubiquitylating activity stimulates its turnover through proteasome, and so deubiquitylation is required for the maintenance of Mib. Therefore, Usp1, Usp9 and Trabid are potential candidates in regulating the stability of Mib/Mib2. However, whether the interaction between Mib/Mib2 and these DUBs is related to Notch signaling remains unclear. Of notice, another two DUBs, eIF3f and Bap1, have been shown in regulating Notch activation [35] and implicated in participating in Notch signaling [36]–[37], respectively.

Tumor necrosis factor (TNF)-α and interleukin (IL)-1 are pro-inflammatory cytokines that initiate signaling pathways in endothelial cells leading to activation of nuclear factor κB (NF-κB) and plays an important role in the regulation of immune and inflammatory responses [38]–[39]. NF-κB activation through IL-1 requires several adaptors, including tumor-necrosis-factor-receptor-associated factor 6 (TRAF6) [40]. Human TRAF-binding domain (TRABID) was able to interact with TRAF6 [39], suggesting a role of Trabid in regulating NF-κB activation.

### Notch and hypoxia signaling pathways

The regulation of hypoxia signaling pathway is conducted by O_2_ sensing enzymes, such as HIF-α-specific prolyl hydroxylase domain proteins (PHDs) and Hif1an/Fih-1, an asparaginyl hydroxylase. FIH-1 interacts with and mediates hydroxylation of HIF-1α and, therefore, inhibits HIF-1 transcriptional activity [41]–[42].

Recently, Fih-1 was found that it can associate with and hydroxylate proteins containing AND, such as Notch receptor 1, 2 and 3 [43], IκBα [44], and Tankyrase-2, Rabankyrin-5 and RNase L [45]. At hypoxia, NICD enhances recruitment of HIF-1α to its target promoters and derepresses HIF-1α function by sequestering HIF-1α through its higher affinity to FIH-1 [30]. Moreover, the crosstalks between hypoxia and Notch signaling pathways were also found to be critical in stem cell maintenance [21], [46], arterial cell fate decision [47], tumor invasion [48], blood cell survival [49] and angiogenesis [50]. Although these lines of evidence indicate a close cooperation between Notch and hypoxia molecules, the interaction of Mib, a critical Notch signaling molecule, and Fih-1, an important hypoxia sensor, was first documented here.

Despite the fact that Mib was not identified in a previous proteomics-based screen for FIH substrates [45], we found that Mib and Mib2 were associated with Fih-1 in both yeast-two hybrid and co-immunoprecipitation experiments. This suggests that Mib/Mib2 and Fih-1 may regulate hypoxia signaling pathway and Notch signaling pathway through each other. The function of Fih-1 on Mib is probably to stabilize the folding structure of Mib, as Fih-1 was found to stabilize the folding of AND through hydroxylation [51], and, thereby, further enhance Notch activation. Mib may facilitate Fih-1 ubiquitylation and degradation as Siah-1, also an E3 ligase, does [52]–[53].

### Mib and Mib2 in cellular trafficking

Enodcytosis of ligands is required for the activation of Notch signaling [54]. Ligands ubiquitylated by E3 ligase Neuralized or Mib are recognized as cargos by the endocytic adaptor Epsin that initiates the Clathrin/Dynamin dependent endocytosis. So far, it is believed that the major role of Mib/Mib2 in Notch signaling is to ubiquitylate ligands. However, fewer studies work on the possible role of Mib/Mib2 in endocytosis. In fact, substrates of Mib/Mib2, such as Delta and RYK, were translocated to the intracellular vesicles with Mib/Mib2 [10], [25]. Results from our screens showed that trafficking molecules like EPS15 and Epsin-2-like are possible binding proteins of Mib2. What are their roles? One possibility is that the interaction of EPS15 and Epsin-2-like with Mib2 is an advantage for forming endocytotic structure immediately after ligands are ubiquitylated by Mib2. Another possibility is that Mib2 ubiquitylates EPS15 and Epsin-2-like to regulate the endocytosis directly. Monoubiquitylation of EPS15 and Epsin is known as a negative regulation to inhibit their binding activity with ubiquitylated cargo on the cell membrane [55]–[56]. The study in fly *Drosophila* indicates that Fat facets (Usp9 ortholog) promotes endocytosis of Delta by deubiquitylating Liquid facets (Epsin ortholog) [57]–[58]. In addition, we have demonstrated Usp9 is an interacting protein of Mib2 by the yeast two-hybrid screen and a binding protein of Mib and Mib2 by immunoprecipitation, which is consistent with previous study by affinity purification/tandem mass spectrometry - Fat facets in Mouse (FAM) or mUSP9x interacts with Mib1 [59]. Therefore, we postulate that Mib and Mib2 may cooperate with Usp9 in regulating endocytosis of Delta through the ubiquitylation/deubiquitylation of Epsin.

Mib may also participate in other trafficking processes. Trafficking molecules like exocyst complex component 1, exocyst complex component 3, and vesicle docking protein p115 were found in our screens. Similarly, other trafficking components have also been identified by other group, such as Importin alpha and beta, Kinesin heavy chain member 2, Nipsnap1, Rba11-F1p2, Rab11-FIP5 and early endosome antigen 1 [59]. These findings imply that trafficking functions of Mib/Mib2 are worthy of further exploration.

### *Status quo* and perspectives

It has been reported that yeast protein interactomes obtained from different research groups by large-scale two-hybrid screens fail to overlap to a great extent [60]–[61]. Reasons ranging from methodology, plasmid constructs, strategy, stringency, saturation and stochastic activation of reporter genes have been discussed. Similarly, these reasons can explain why our screens did not pick up the genes identified by other groups (e.g., DSL ligands, listed in Table 3). The ubiquitylation of Mib-interacting proteins affects their localization (e.g., Jagged 1, Jagged 2, DeltaC, DeltaD, CDK5, Snx5 and RYK), protein stability (e.g., DAPK, RYK and SMN) or activity (e.g., TBK1 and NMDAR) [9]–[12], [23]–[25], [59], [62]–[65]. Similar to Mib, Mib2 can regulate the localization (e.g., DeltaC and Jagged 2) and activity (e.g., TAK1) of its interacting proteins [10], [66]–[67]. Additionally, another Mib-interacting protein PAR-1 can phosphorylate Mib. This results in Mib degradation and, therefore, suppresses Notch signaling and stimulates neuronal differentiation [68]. Most importantly, our screens complement previous studies and add novel interacting proteins to an expanding list (Table 3) for further exploration of Mib/Mib2 functions.

**Table 3:** The confirmed interacting proteins of Mind bomb and Mind bomb 2.

Interacting protein	Mib/Mib2	Method	Effects on interacting proteins	Function	Reference
Death associated protein kinase (DAPK)	Mib	Yeast-two-hybrid	Ubiquitylation, Proteasomal degradation	Antagonize the anti-apoptotic function of DAPK	[23]
Cellular Fas-associated death domain -like IL-1b converting enzyme-like inhibitory protein (cFLIP)	Mib	IP	Caspase-dependent degradation	Decrease the association of caspase-8 and cFLIP, leading to cell apoptosis	[24]
Jagged 1a and 1b (Jag1a and 1b)	Mib	Yeast-two-hybrid	Ubiquitylation	Activate the Notch signaling pathway	[11]
Jagged 2 (Jag2)	Mib, Mib2	IP, GST pulldown	Internalization, Ubiquitylation	Activate the Notch signaling pathway	[12], [66]
xDelta 1	Mib	IP	Ubiquitylation, Internalization	Activate the Notch signaling pathway	[9]
DeltaA	Mib	IP	Predicted: Ubiquitylation, Internalization	Activate the Notch signaling pathway	[10]
DeltaC	Mib, Mib2	IP	Ubiquitylation, Internalization	Activate the Notch signaling pathway	[10]
DeltaD	Mib	IP	Ubiquitylation, Internalization	Activate the Notch signaling pathway	[9], [10]
Mind bomb 2 (Mib2)	Mib	IP	Ubiquitylation	Predicted: Activate the Notch signaling pathway	[15]
Sorting nexin 5 (Snx5)	Mib	Yeast-two-hybrid	unknown	Predicted: Mind bomb-mediated endocytosis of Notch ligand proteins	[64]
TANK-binding kinase 1 (TBK1)	Mib	Mass spectrometry	K63-linked ubiquitylation activation	Regulate innate immune responses to RNA virus infection	[62]
Cyclin-dependent kinase 5 (CDK5)	Mib	Mass spectrometry	Localization	Inhibit neurite outgrowth	[59]
PAR-1	Mib	Mass spectrometry	Phosphorylation of Mib, leading to the proteasomal degradation of Mib	Repress the Notch signaling pathway and stimulate neuronal differentiation	[68]
Receptor-like tyrosine kinase (RYK)	Mib	Mass spectrometry	Ubiquitylation, Proteasomal degradation, Internalization	Activate the Wnt signaling pathway	[25]
Survival of motor neuron protein (SMN)	Mib	IP	Ubiquitylation, Proteasomal degradation	A therapeutic candidate for the spinal muscular atrophy	[63]
B-cell CLL/lymphoma 10 (BCL10)	Mib2	Mass spectrometry	unknown	Activate the NF-κB signaling pathway	[67]
Inhibitor of nuclear factor kappa-B kinase subunit gamma (IKKγ)	Mib2	IP	Ubiquitylation	Activate the NF-κB signaling pathway	[67]
Transforming growth factor-β-activated kinase 1 (TAK1)	Mib2	IP	Activation	Activate the NF-κB signaling pathway	[67]
*N*-methyl-D-aspartate receptor (NMDAR)	Mib2	Yeast-two-hybrid	Ubiquitylation	Reduce the NMDAR activity	[65]
Ubiquitin specific protease 1 (Usp1)	Mib, Mib2	Yeast-two-hybrid, IP	unknown	unknown	this study
Ubiquitin specific protease 9 (Usp9)	Mib, Mib2	Yeast-two-hybrid, IP	unknown	unknown	this study
Tumor-necrosis-factor-receptor-associated factor-binding domain (Trabid)	Mib, Mib2	Yeast-two-hybrid, IP	Ubiquitylation	unknown	this study
Factor inhibiting HIF 1 (Fih-1)	Mib, Mib2	Yeast-two-hybrid, IP	Ubiquitylation	unknown	this study

In our screens, several proteins involved in the cytoskeleton, including actinin alpha 2, actinin alpha 3, actinin alpha 4, myosin light chain and cofilin 1, were identified. Cytoskeleton member like dystrobrevin and dystrophin-associated protein A1 were also identified as Mib-interacting proteins by affinity purification and mass spectrometry [59]. Whether Mib participates in the muscle development or maintenance is unclear. Studies in *Drosophila* indicated that Mib2 expressed in founder myoblasts regulates myoblast fusion and muscle stability through a Notch-independent way [69]–[70]. Although no evidence supports the involvement of Mib in *Drosophila* myogenesis, the putative Mib-interacting proteins related to cytoskeleton do implicate such a role in vertebrates.

Proteins involved in cell signaling also appear in our screens, such as polo-like-kinase 4, tyrosine 3-monooxygenase/tryptophan 5-monooxygenase activation proteins (14-3-3), TGF-beta activated kinase 1/MAP3K7 binding protein 3 (TAK1-binding protein 3), creatine kinase, and casein kinase 2 beta (ck2b), suggesting that Mib/Mib2 are potentially involved in several signaling pathways in addition to the Notch signaling pathway. Indeed, Mib plays a positive role in the Wnt signaling pathway by regulating the stability and localization of RYK [25]. Mib2 activates NF-κB pathway through the interaction with B-cell CLL/lymphoma 10 (BCL10), inhibitor of nuclear factor kappa-B kinase subunit gamma (IKKγ) and transforming growth factor β-activated kinase 1 (TAK1) [67]. In the past, the roles of Mib in the neurogenesis, gliogenesis, thyroid morphogenesis, T helper cell differentiation and pancreatic β-cell formation were focused on the Notch-dependent pathway [71]–[74]. In the future, it is rational to take other potential cell signaling pathways into consideration for a more deep understanding of the roles of Mib and Mib2 in various developmental processes.

## Materials and Methods

### Yeast two-hybrid screen

For the first yeast two-hybrid screen, a conventional LexA and Gal4 system relying on transcriptional activation of reporter genes in the nucleus to detect interactions was used. Bait fragments used for this screen were Mib-N+Ank (1–782 aa), Mib-C-RF123a (783–1029 aa), Mib2-N+Ank (54–780 aa) and Mib2-C-RF12 (781–999 aa) (Figure 1B). Yeast two-hybrid screening was performed by Hybrigenics (Paris, France). The corresponding fragments of *mib* and *mib2* [15] was PCR-amplified and cloned into pB27 plasmid for N-terminal LexA DNA binding domain fusion proteins (N-LexA-Mib/Mib2-C). The constructs were checked by sequencing and used as baits to screen a random-primed *Danio rerio* embryo (stages 18–20 hpf) RP1 cDNA library (Hybrigenics) constructed into pP6 plasmid for C-terminal Gal4 transcription activating domain fusion proteins. Mib-N+Ank (1–782 aa) and Mib2-N+Ank (54–780 aa) have autoactivation effect and, therefore, are not used for further screen. 60 million colonies for Mib-C-RF123a (783–1029 aa) and 52 million colonies for Mib2-C-RF12 (781–999 aa) were respectively screened. cDNA fragments corresponding to positive “prey” clones were amplified by PCR and sequenced at their 5′ and 3′ junctions. The resulting sequences were searched against GenBank using a fully automated procedure, assigned a quality score (PBS, for Predicted Biological Score) indicative of the confidence of interaction [75] and listed in Table 1.

For the second yeast two-hybrid screen, zebrafish Mib-N (1–462 aa), Mib-M-Ank (454–785 aa), Mib-C-RF123b (789–1030 aa) and Mib-ZnF+Ank (74–785 aa) (Figure 1B) were subcloned into pSos vector (Stratagene) as baits by PCR amplification of *mib* cDNA sequences (using BamHI and XhoI as cloning sites). These baits were then used to screen the prey, pMyr-cDNA library (CytoTrap® XR Zebrafish Embryo Plasmid cDNA Library, Stratagene; normal, whole, zebrafish embryos, average insert size: 1.0 kb) within the temperature-sensitive yeast mutant strain cdc25H according to the manufacturer's instructions (Stratagene). *cdc25* is the yeast homolog of the human *Sos* (*hSos*) gene, which encodes a guanyl nucleotide exchange factor that activates Ras signal transduction pathway. The *cdc25* mutation present in the cdc25H strain allows normal growth at the permissive temperature (25°C), but prevents growth at 37°C. pSos bait vector generates a fusion protein of hSos and the bait protein. pMyr-cDNA is expressed as a fusion protein with a myristylation sequence that anchors the fusion protein to the plasma membrane. The physical interaction of the bait and prey proteins results in the localization of hSos to the plasma membrane and reconstitutes Ras-signaling pathway activity, which allows the yeast growth at both 37°C and 25°C. pSos-Mib-N (1–462 aa) was unsuitable for detecting protein-protein interactions in the CytoTrap system, because the bait plasmid cotransformed with the pMyr empty vector autoactivated Ras signal and induced cdc25H yeast growth at 37°C. The screen then focused on the other three baits. Around 0.4, 0.5 and 0.5 million colonies were screened for Mib-M-Ank (454–785 aa), Mib-C-RF123b (789–1030 aa) and Mib-ZnF+Ank (74–785 aa), respectively. Plasmid DNA was isolated from the putative positive pMyr-cDNA clones. The two-hybrid interaction was confirmed by retransformation of the yeast strain cdc25H with the bait vector and purified prey plasmid. All positive prey plasmids were sent for DNA sequencing by the 5′ primer (5′-ACTACTAGCAGCTGTAATAC-3′) in the pMyr vector and blasted again NCBI database. Clones that have in-frame cDNA sequence with the myristylation sequence were finally selected and listed in Table 2.

### Plasmids

pCS2-FLAG-*mib* and -*mib2* were made by subcloning the PCR-amplified fragments into the pCS2+FLAG vector [10]. Full-length *fih-1* and partial *trabid C* (295–716 aa) cDNA fragments were PCR-amplified and subcloned into pCS2-MT vector at EcoRI and XhoI sites. Full-length *trabid* cDNA fragments were PCR-amplified and subcloned into pCS2-MT vector at XhoI and XbaI sites. C-terminal *usp1 C* (349–772 aa) cDNA fragments were PCR-amplified and subcloned into pCS2-MT vector at EcoRI and XhoI sites. Full-length *usp9* cDNA fragment was PCR-amplified and subcloned into pCMV-HA vector at SfiI and NotI sites. All PCR products were verified by DNA sequencing. pcDNA3.1-HA-*ubiquitin* is a gift from Dr. Margaret A. Shipp. Usp1, Usp9, Trabid and Fih-1 protein sequences are shown in Supporting Information with comparison to those of other species (Figures S1–S4).

### Cell culture and co-immunoprecipitation assay

COS7 cells [10], [15] were cultured in Dulbecco's modified Eagle's medium (Gibco) supplemented with 10% (v/v) fetal bovine serum and 1% penicillin-streptomycin. Cells were transiently transfected using Lipofectamine 2000 transfection reagent (Invitrogen). 4 µg of pCS2-MT-*fih-1* was cotransfected with 4 µg of pCS2-FLAG-*mib* or -*mib2* into COS7 cells. 2 µg of pCS2-MT-*trabid C* (295–716 aa), pCS2-MT-*usp1* (349–772 aa) or pCMV-HA-*usp9* was cotransfected with 2 µg of pCS2-FLAG-*mib* or -*mib2* into COS7 cells. Cells were harvested and lysed 24 h after transfection in IONIC buffer (50 mM Tris HCl [pH 7.4], 150 mM NaCl, 1% Triton X-100, containing a protease inhibitor cocktail, Calbiochem). The cell lysate was clarified by centrifugation and incubated with anti-FLAG M2 affinity gel (Sigma) for 2 h at 4°C. The gel was boiled in SDS gel loading buffer and the eluted proteins were electrophoresed on a SDS-PAGE, and transferred to a polyvinylidene difluoride membrane (Millipore). Blots were incubated with primary antibody: anti-HA (Protech), anti-Myc (Santa Cruz) or anti-FLAG (Sigma) for 2 hr. The signal was visualized using a secondary antibody: HRP-conjugated anti-mouse IgG antibody (GeneTex) or HRP-conjugated anti-rabbit IgG antibody (GeneTex), with a chemiluminescence detection system (PerkinElmer Inc).

### *In vivo* substrate ubiquitylation assay

COS7 cells were transiently transfected with 2 µg of pcDNA3.1-HA-*ubiquitin*, 2 µg of pCS2-MT-*fih-1* [or pCS2-MT-*trabid C* (295–716 aa)] as substrate and 4 µg of pCS2-FLAG-*mib* or -*mib2* as putative E3 ubiquitin ligases. 2 µM proteasome inhibitor MG132 was added to the cell culture medium after 24 h. Cells were harvested at 48 h and lysed in IONIC buffer and 10 µM MG132. The cell lysate was clarified by centrifugation and incubated with rabbit anti-Myc antibody (Santa Cruz) for 2 h at 4°C, and then incubated with protein A Sepharose beads for 1 h at 4°C. The eluted proteins were electrophoresed on a SDS-PAGE followed by Western blotting with mouse anti-HA (Roche) or mouse anti-Myc (Roche) antibody.

## Supporting Information

Figure S1 **Alignment of Usp1 amino acid sequences.** Alignment of zebrafish Usp1 (*Danio rerio*, NP_955873.1), chicken Usp1 (*Gallus gallus*, NP_001026461.1), mouse Usp1 (*Mus musculus*, AAH20007.1) and human Usp1 (*Homo sapiens*, NP_001017416.1). Zebrafish Usp1 bears 51.49%, 52.39% and 53.14% amino acids identity to its counterparts of chicken, mouse and human, respectively. Red line indicates the region of ubiquitin-specific protease (USP) domain.
(PDF)

Figure S2 **Alignment of Usp9 amino acid sequences.** Alignment of zebrafish Usp9 (*Danio rerio*, our sequence), human Usp9 (*Homo sapiens*, XP_005272733.1) and chicken Usp9 (*Gallus gallus*, XP_416773.2). Zebrafish Usp9 bears 90.04% and 92.58% amino acids identity to its counterparts of human and chicken, respectively. Red line indicates the region of ubiquitin-specific protease (USP) domain.
(PDF)

Figure S3 **Alignment of Trabid amino acid sequences.** Alignment of zebrafish Trabid (*Danio rerio*, our sequence), African clawed frog Trabid (*Xenopus laevis*, NP_001084698), human Trabid (*Homo sapiens*, CAB64449) and mouse Trabid (*Mus musculus*, NP_997185). Zebrafish Trabid bears 78.88%, 80.17% and 80.31% amino acids identity to its counterparts of *Xenopus*, human and mouse, respectively. Blue line indicates the region of Npl4 zinc finger domain (NZF). Green line indicates the region of ankyrin repeat. Red line indicates the region of ovarian tumor protease (OTU) domain.
(PDF)

Figure S4 **Alignment of Fih-1 amino acid sequences.** Alignment of zebrafish Fih-1 (*Danio rerio*, our sequence), human Fih-1 (*Homo sapiens*, NP_060372.2) and mouse Fih-1 (*Mus musculus*, NP_795932.2). Zebrafish Fih-1 bears 81.76% and 82.35% amino acids identity to its counterparts of human and mouse, respectively. Purple line indicates the region of Jmjc domain.
(PDF)
