# Supplementary material for: New Classes of Mind Bomb-Interacting Proteins Identified from Yeast Two-Hybrid Screens
Source: PLoS One. 2014 Apr 8;9(4):e93394. doi: 10.1371/journal.pone.0093394 (PMC3979679; doi:10.1371/journal.pone.0093394)
Supplement: Figure S1 — Alignment of Usp1 amino acid sequences. Alignment of zebrafish Usp1 (Danio rerio, NP_955873.1), chicken Usp1 (Gallus gallus, NP_001026461.1), mouse Usp1 (Mus musculus, AAH20007.1) and human Usp1 (Homo sapiens, NP_001017416.1). Zebrafish Usp1 bears 51.49%, 52.39% and 53.14% amino acids identity to its counterparts of chicken, mouse and human, respectively. Red line indicates the region of ubiquitin-specific protease (USP) domain. (PDF) [file pone.0093394.s001.pdf]

Figure S1. Alignment of Usp1 amino acid sequences

```
1    MPGLHSESGVVAAAGSPVKKSKLSLKFFQKKETKRALDFSEAPPEDNSNVTTEPQETICHDQVVPLPCPSSPLTCEKRESLVPFVGLNNLGNTCYLNSIL  zebrafish
1    MPGVLPSDGSAPPRGSPSRKNRLSLKLFQKKEAKRALDFTEPPENEQKGAE---LRGAEIDQVVPAAQPPSLVSCEKKDNMLPFVGLNNLGNTCYLNSVL  chicken
1    MPGVIPSESNGLSRGSPSKKNRLSLKFFQKKETKRALDFTDSQENEEKTSE---YRGSEIDQVVPAAQS-SPVSCEKRENLLPFVGLKNLGNTCYLNSIL  mouse
1    MPGVIPSESNGLSRGSPSKKNRLSLKFFQKKETKRALDFTDSQENEEKASE---YRASEIDQVVPAAQS-SPINCEKRENLLPFVGLNNLGNTCYLNSIL  human

101  QVLYYCPGFKEAIKSLCHVAKLKDKPQEEGVNNEGDSG--ENTLPVPMELLGSFHSLISSVEQLQSSFLLNPEKYNDGELATPPRKLLNALRQLNPMYEG
98   QVLYFCPGFKTGVKHLYNIISKKKESLKDEGELKAEKGNCKEDPLASYELICSLHSLILSVEQLQASFLLNPEKYTD-ELATQPRRLLNTLRELNPMYEG
97   QVLYFCPGFKTGVKHLFNIISRKKEALKDDSNQ-KDKGSCKEESLASYELICSLQSLIISVEQLQASFLLNPEKYTD-ELATQPRRLLNTLRELNPMYEG
97   QVLYFCPGFKSGVKHLFNIISRKKEALKDEANQ-KDKGNCKEDSLASYELICSLQSLIISVEQLQASFLLNPEKYTD-ELATQPRRLLNTLRELNPMYEG

199  YLQHDAQEVLQCILVNIQEACDTIKKEQTDNQDTTMING--------------TESVQ-----------DGEG--SSDSQLSGKRKSDTEAGNAKKKPKS
197  YLQHDAQEVLQCILGNIQETCQLLKKEELNKLPLEEPKAKVEEKINPNSESNGTGNSAEEEEENAPGSHVGEVAEDKLPKGNGKRKSDAEGGNAKKKSKV
195  FLQHDAQEVLQCILGNIQETCQLLKKEEIKNLAE--LSGKVEEQSLQKEETGGITSTEI------DSMRNTEDVKEQLPKGNWKRKSDSESSNVKKKVKL
195  YLQHDAQEVLQCILGNIQETCQLLKKEEVKNVAE--LPTKVEEIPHPKEEMNGINSIEM------DSMRHSEDFKEKLPKGNGKRKSDTEFGNMKKKVKL

272  QSKSKKNEENVPMTRSKRKSSSEISTENSATEQRNGTEEQEKDSTTEEEKNDSPPKDTGKRTRRGKLGWLRPSGKQPSIFSKFRSMGRITSHVGAKGETK
297  SKEQIAAEENQRQTRSKRKATGEKLENQSDAIAKC--------------STENESAKPTQKKSRLKLNWLKSSCKQPSILSKFYSLGRLSTNLGSKD-PE
287  SRESQPLEENQRQTRSKRKAIGDTLEAAPKIIPKC--------------VSESESAKPSQKKSKVKINWLKSSTKQPSILSKFCSLGKITTNQRSKGQPK
287  SKEHQSLEENQRQTRSKRKATSDTLESPPKIIPKY--------------ISENESPRPSQKKSRVKINWLKSATKQPSILSKFCSLGKITTNQGVKGQSK

372  EKSDCSSQEKQDKDNSENEN----------------KTHEVKQDLGKNEEQSGLDVLKWMFQGQLVLRTRCLECECFTERREDFQDISVPVQEDKTLSSD
382  YDD-CELEESSAKCENGNDIKEEDCHEPASPVESHTEKGTEKEPKNEGTELAVFELVEKLFQGQLVLRTRCLECECFTERREDFQDISVPVQEDELSKTE
373  EKE-GDVEEDLEKYGSDHTANG-GPESPGSSV--TPVDSSEAKSGNKGAEQIGFELVEKLFQGQLVLRTRCLECESLTERREDFQDISVPVQEDELSKVE
373  ENE-CDPEEDLGKCESDNTTNGCGLESPGNTV--TPVNVNEVKPINKGEEQIGFELVEKLFQGQLVLRTRCLECESLTERREDFQDISVPVQEDELSKVE

456  SSSEISPDPKTELKTLKWAISQFASVERIVGQDKYFCETCHHYTEAERSLLFDKTPEVITIHLKCFAANGSEMDPYA-GLSKVNTPLQTPLKLSLHEWCT
481  ESSEISPEPKTEMKTLKWAISQFASVERIVGEDKYFCENCHHYTEAERSLLFDKMPEVITIHLKCFAASGLEFDCYG-GLSKINTPLLTPLKLSLEEWST
469  ESSEISPEPKTEMKTLRWAISQFASVERIVGEDKYFCENCHHYTEAERSLLFDKMPEVITIHLKCFAASGLEFDCYGGGLSKINTPLLTPLKLSLEEWST
470  ESSEISPEPKTEMKTLRWAISQFASVERIVGEDKYFCENCHHYTEAERSLLFDKMPEVITIHLKCFAASGLEFDCYGGGLSKINTPLLTPLKLSLEEWST

555  QPDSPDYELFAVVMHSGVTISSGHYTTYIRMMDLHHTNIKPQTQ--DEEHDRDQEEDMKQKKEETSQTDYDDGEVSFSLSGRGRNVARTSGTNNMSSKTA
580  KPTNDTYGLFAVVMHSGITISSGHYTASVKITDLNSLELDKGNFITAQMCETIKPEPLNEEEARTAAEDYDDGEVSFRVNGNA-----------QPGKVL
569  KPTNDSYGLFAVVMHSGITISSGHYTASVKVTDLNSLELDEGNFVVDQMCELGKPEPLTEEQARGTAGNYDD-EVSIRVGGNA-----------QPSKVL
570  KPTNDSYGLFAVVMHSGITISSGHYTASVKVTDLNSLELDKGNFVVDQMCEIGKPEPLNEEEARGVVENYNDEEVSIRVGGNT-----------QPSKVL

653  GKRCSEGVGLLGGQRSITSYELSNSKQNNPDKPSSLSSRALLQNAVK--------------KEPEEDSADAVADGTQVSFDLALRNLLDFEGKWMLFDDS
669  NKKNMEAVGLLGGQKSKSDCDLYSKPAS-SEKVLNI---V-TENRNPESAPA-NG--SVECSTERGDQNGVAFSGLENKALYVLQSLKEYEGKWLLFDDS
657  NKKNVEGIGLLGGQKSKADYELYNKASN-PDKVVGT---PFTDNRNSETNDTTNGTHESDRNKESSDQTGVNMNGLENKISYVVQSLKEYEGKWLLFDDS
659  NKKNVEAIGLLGGQKSKADYELYNKASN-PDKVAST---AFAENRNSETSDT-TGTHESDRNKESSDQTGINISGFENKISYVVQSLKEYEGKWLLFDDS

739  EVRLYEEEDFLRACSPETCSTSTPYLLFYKRVSQ
761  EVKVTEEKDFLNSLSPTSSSTSTPYLLFYKKIVE
753  EVKVTEEKDFLNSLSPSTSPTSTPYLLFYKKL--
754  EVKVTEEKDFLNSLSPSTSPTSTPYLLFYKKL--
```

Figure S1. Alignment of Usp1 amino acid sequences. Alignment of zebrafish Usp1 (*Danio rerio*, NP_955873.1), chicken Usp1 (*Gallus gallus*, NP_001026461.1), mouse Usp1 (*Mus musculus*, AAH20007.1) and human Usp1 (*Homo sapiens*, NP_001017416.1). Zebrafish Usp1 bears 51.49%, 52.39% and 53.14% amino acids identity to its counterparts of chicken, mouse and human, respectively. Red line indicates the region of ubiquitin-specific protease (USP) domain.
