# Supplementary material for: New Classes of Mind Bomb-Interacting Proteins Identified from Yeast Two-Hybrid Screens
Source: PLoS One. 2014 Apr 8;9(4):e93394. doi: 10.1371/journal.pone.0093394 (PMC3979679; doi:10.1371/journal.pone.0093394)
Supplement: Figure S2 — Alignment of Usp9 amino acid sequences. Alignment of zebrafish Usp9 (Danio rerio, our sequence), human Usp9 (Homo sapiens, XP_005272733.1) and chicken Usp9 (Gallus gallus, XP_416773.2). Zebrafish Usp9 bears 90.04% and 92.58% amino acids identity to its counterparts of human and chicken, respectively. Red line indicates the region of ubiquitin-specific protease (USP) domain. (PDF) [file pone.0093394.s002.pdf]

Figure S2. Alignment of Usp9 amino acid sequences

```
1     MTATTRGSPVGGNDGQGQAPDGQSQPPLPQNQTSSPNSSNENSPVSPPDDQGQGDSPTPLEEEEPAFPHTELAKLDDMINRPRWVVPVLPKGELEVLLEA  zebrafish
1     MTATTRGSPVGGNDNQGQAPDGQSQPPLQQNQTSSPDSSNENSPATPPDEQGQGDAPPQLEDEEPAFPHTDLAKLDDMINRPRWVVPVLPKGELEVLLEA  human
1     MTATTRGSPVGGNDSQGQAPDGQSQPPLQQNQTSSPDSSNENSPATPPDEQGQGDAPPQLEDEEPAFPHTDLAKLDDMINRPRWVVPVLPKGELEVLLEA  chicken

101   AIDLCKKGIDVKCEACQRFFRDGLTISFTKILTDEAVSGWKFEIHRCIINNAHRLVELCVTKLSQDWFPLLELLAMATNPHCKFHIYNGTRPSETVPAGV
101   AIDLSKKGLDVKSEACQRFFRDGLTISFTKILTDEAVSGWKFEIHRCIINNTHRLVELCVAKLSQDWFPLLELLAMALNPHCKFHIYNGTRPCESVSSSV
101   AIDLSKKGLDVKSEACQRFFRDGLTISFTKILTDEAVSGWKFEIHRCIINNTHRLVELCVAKLSQDWFPLLELLAMALNPHCKFHLYNGTRPSETVPAGV

201   QLAEDELFARPPDPRSPKGWLVDLINKFGTLNGFQTLHDRFMSGQALNVQIIAALIKPFGQCYEFLTLHTVKKYFLPIIEMVPQFLENLTDEELKKEAKN
201   QLPEDELFARSPDPRSPKGWLVDLLNKFGTLNGFQILHDRFINGSALNVQIIAALIKPFGQCYEFLTLHTVKKYFLPIIEMVPQFLENLTDEELKKEAKN
201   QLAEDELYARPPDPRSPKGWLVDLINKFGTLNGFQILHDRFMSGSALNVQIIAALIKPFGQCYEFLTLHTVKKYFLPIIEMVPQFLENLTDDELKKEAKN

301   EAKNDALSMIIKSLKNLASRVPGQEETVKNLEIFRLKMILRLLQISSFNGKMNALNEVNKVISSVSYYTHRHGNPEEEEWLTAERMAEWIQQNNILSIVL
301   EAKNDALSMIIKSLKNLASRVPGQEETVKNLEIFRLKMILRLLQISSFNGKMNALNEVNKVISSVSYYTHRHGNPEEEEWLTAERMAEWIQQNNILSIVL
301   EAKNDALSMIIKSLKNLASRVPGQEETVKNLEIFRLKMILRLLQISSFNGKMNALNEVNKVISSVSYYTHRHGNPEEEEWLTAERMAEWIQQNNILSIVL

401   RDSLHQPQYVEKLEKILRFVIKEKALTLQDLDNIWAAQAGKHEAIVKNVHDLLAKLAWDFSPEQLDHLFDCFKESWTNASKKQREKLLELIRRLAEDDKD
401   RDSLHQPQYVEKLEKILRFVIKEKALTLQDLDNIWAAQAGKHEAIVKNVHDLLAKLAWDFSPEQLDHLFDCFKASWTNASKKQREKLLELIRRLAEDDKD
401   RDSLHQPQYVEKLEKILRFVIKEKALTLQDLDNIWAAQAGKHEAIVKNVHDLLAKLAWDFSPEQLDHLFDCFKASWTNASKKQREKLLELIRRLAEDDKD

501   GVMAHKVLNLLWNLAHSDDVPVDIMDQALSAHIKILDYSCSQDRDTQKMQWIDRFIEELRTNDKWVIPALKQIREICSLFGEAPQNLS-----QTQRSPH
501   GVMAHKVLNLLWNLAHSDDVPVDIMDLALSAHIKILDYSCSQDRDTQKIQWIDRFIEELRTNDKWVIPALKQIREICSLFGEAPQNLSSSRFSQTQRSPH
501   GVMAHKVLNLLWNLAHSDDVPVDIMDQALSAHIKILDYSCSQDRDTQKIQWIDRFIEELRTNDKWVIPALKQIREICSLFGEAPQNLS-----QTQRSPH

596   VFYRHDLINQLQHNHALVTLVAENLSAYMENMRQFSKEHADFDPQTVRPGSRYSHVQEVQERLNFLRFLLKDGQLWLCAPQAKQIWKCLAENAVFLCDRE
601   VFYRHDLINQLQHNHALVTLVAENLATYMESMRLYARDHEDYDPQTVRLGSRYSHVQEVQERLNFLRFLLKDGQLWLCAPQAKQIWKCLAENAVYLCDRE
596   VFYRHDLINQLQHNHALVTLVAENLSAYMESMRQYAKEHGEYDPQTVRPGSRYSHVQEVQERLNFLRFLLKDGQLWLCAPQAKQIWKCLAENAVYLCDRE

696   ACFKWYSKLMGDEPDLDPDINKDFFENNVLQLDPSLLTENGMKCFERFFKAVNCREGKLVAKRRAYMMDDLELIGLDYLWRVVIQGSDDIASRAIDLLKE
701   ACFKWYSKLMGDEPDLDPDINKDFFESNVLQLDPSLLTENGMKCFERFFKAVNCREGKLVAKRRAYMMDDLELIGLDYLWRVVIQSNDDIASRAIDLLKE
696   ACFKWYSKLMGDEPDLDPDINKDFFENNVLQLDPSLLTENGMKCFERFFKAVNCREGKLVAKRRAYMMDDLELIGLDYLWRVVIQGNDDIANRAIDLLKE

796   IYTNLGPKLQANQVEIHEDFIQSCFDRLKASYDTLCVLDGDKDSINCARQEAIRMVRVLTVLREYITECDSDYHEERTILPMSRAFRGKHITLVVRFPNQ
801   IYTNLGPRLQVNQVVIHEDFIQSCFDRLKASYDTLCVLDGDKDSVNCARQEAVRMVRVLTVLREYINECDSDYHEERTILPMSRAFRGKHLSFVVRFPNQ
796   IYTNLGPRLQVNQVVIHEDFIQSCFDRLKASYDTLCVLDGDKDSINCARQEAIRMVRVLTVLREYINECDSDYHEERTILPMSRAFRGKHITLVVRFPNQ

896   GRQVDDLDIWSHTNDTIGSVRRCILNRIKANSTHTKIELFIGGEIIDPADDRKLIGQLNLKDKTLITAKLTQVSANMPSSPDSSSDSSTGSPGNHGNHYS
901   GRQVDDLEVWSHTNDTIGSVRRCILNRIKANVAHTKIELFVGGELIDPADDRKLIGQLNLKDKSLITAKLTQISSNMPSSPDSSSDSSTGSPGNHGNHYS
896   GRQVEDLDIWSHTNDTIGQVRRCILNRIKANSAHTKVELFIGGELVDPADDRKLIGQLNLKDKTLITAKLTQINSNMPSSPDSSSDSSTGSPGNHGNHYS

996   DGPNPEVESCLPGVIMSLHVRYISFLWQVADLGCNLNMPLLRDGARVLMKLMPPDNATVENLRAICLDHAKLGENSLSPTLDSRFFGPSPSQVLYLIEVV
1001  DGPNPEVESCLPGVIMSLHPRYISFLWQVADLGSSLNMPPLRDGARVLMKLMPPDSTTIEKLRAICLDHAKLGESSLSPSLDSLFFGPSASQVLYLTEVV
996   DGPNPEVESCLPGVIMSLHPRYISFLWQVADLGSSLNMPPLRDGARVLMKLMPPDNTTVEKLRAICLDHAKLGESSLSPSLDSLFFGPSASQVLYLTEVV

1096  YALLMPASGTLGEDASDFQYNFLKSGGLPLVLSMLTRNNFLPNADMETRRGAYLNALKIAKLLLTAVGFGHVKSVAEACQPVVEGTIPVSPINQTTHDQA
1101  YALLMPAGAPLADDSSDFQFHFLKSGGLPLVLSMLTRNNFLPNADMETRRGAYLNALKIAKLLLTAIGYGHVRAVAEACQPGVEGVNPMTQINQVTHDQA
1096  YALLMPASAPLGEDASDFQYNFLKSGGLPLVLSMLTRNNFLPNADMETRRGAYLNALKIAKLLLTAIGYGHVRAVAEACQPVVEGTSTVSPINQATHDQA

1196  LVLQNALQNIPNPSAECMLRNVAIRLAQQISDENFFQASKYIPDICVIRAVQKIVWASGCGSVQHVFSSNEEISKIYEKTNAGNEPDAEDEQVCCEALEV
1201  VVLQSALQSIPNPSSECMLRNVSVRLAQQISDE----ASRYMPDICVIRAIQKIIWASGCGSLQLVFSPNEEITKIYEKTNAGNEPDLEDEQVCCEALEV
1196  VVLQNALQNIPNPTSECMLRNVAIRLAQQISDE----ASKYIPDICVIRAVQKIVWASGCGSVQLVFSSNEEISKIYEKTNAGNEPDMEDEQVCCEALEV

1296  MTLCFALMPTALDALSKEKAWQTFIIDLLLHCQSKSVRQMAQEQFFLMATRCCMGHRPLLFFITLLFTVLGSTAKERAKHAADYFTLLRHLLNYAFNSNI
1297  MTLCFALIPTALDALSKEKAWQTFIIDLLLHCHSKTVRQVAQEQFFLMCTRCCMGHRPLLFFITLLFTVLGSTARERAKHSGDYFTLLRHLLNYAYNSNI
1292  MTLCFALIPTALDALSKEKAWQTFIIDLLLHCHSKIVRQMAQEQFFLMATRCCMGQRPLLFFITLLFTVLGSTARERAKHSGDYFTLLRHLLNYAYNSNI

1396  NLPNAEVLLNNEIDWLKRIRDEVKRTGEPGVEETILEGHIGVTKELLAFQTPEKKFYIGCEKGGASLIKELMDDFLFPASNVYLQYMKSGEFPTEQAIPV
1397  NVPNAEVLLNNEIDWLKRIRDDVKRTGETGIEETILEGHLGVTKELLAFQTSEKKFHIGCEKGGANLIKELIDDFIFPASNVYLQYMRNGELPAEQAIPV
1392  NVPNAEVLLNNEIDWLKRIRDEVKRTGETGVEETILEGHLGVTKELLAFQTPEKKYHIGCEKGGANLIKELIDDFIFPASNVYLQYMRNGELPAEQAIPV

1496  CSTPATINAGFELLVALAIGCVRNLKQIVDTLTDMYYLG-----CEPLTEWEYLPPVGPRPTKGFVGLKNAGATCYMNSVIQQLYMIPPIRNGILAIEGT
1497  CGSPPTINAGFELLVALAVGCVRNLKQIVDSLTEMYYIGTAITTCEALTEWEYLPPVGPRPPKGFVGLKNAGATCYMNSVIQQLYMIPSIRNGILAIEGT
1492  CSSPATINAGFELLVALAVGCVRNLKQIVDTLTEMYYIGTAITTCEALTEWEYLPPVGPRPPKGFVGLKNAGATCYMNSVIQQLYMIPAIRNGILAIEGT
```

```
1591 GSDVDDDMSGDEKQDNESNVDPRDEVFGYQHQFDDKPSLSKSEDRKEYNIGVLRQLQVIFGHLASSRLQYYVPRGFWKQFRLWGEPVNLREQHDALEFFN
1597 GSDVDDDMSGDEKQDNESNVDPRDDVFGYPQQFEDKPALSKTEDRKEYNIGVLRHLQVIFGHLAASRLQYYVPRGFWKQFRLWGEPVNLREQHDALEFFN
1592 GSDVDDDMSGDEKQDNESNVDPRDDVFGYPHQYEDKPALSKTEDRKEYNIGVLRHLQVIFGHLAASRLQYYVPRGFWKQFRLWGEPVNLREQHDALEFFN

1691 SLVDSLDEALKALGHPAMLSKVLGGSFADQKICQGCPHRYECEESFTTLNVDIRNHQNLLDSMEQYVKGDLLEGANAYHCEKCNKKVDTVKRLLIKKLPP
1697 SLVDSLDEALKALGHPAMLSKVLGGSFADQKICQGCPHRYECEESFTTLNVDIRNHQNLLDSLEQYVKGDLLEGANAYHCEKCNKKVDTVKRLLIKKLPP
1692 SLVDSLDEALKALGHPAMLSKVLGGSFADQKICQGCPHRYECEESFTTLNVDIRNHQNLLDSLEQYVKGDLLEGANAYHCEKCNKKVDTVKRLLIKKLPP

1791 VLAIQLKRFDYDWERECAIKFNDYFEFPRELDMEPYTVAGVAKLEGSDVHPENQQQVIQQNEPSEPEPPCSSRYRLVGVLVHSGQASGGHYYSYIIQRNG
1797 VLAIQLKRFDYDWERECAIKFNDYFEFPRELDMEPYTVAGVAKLEGDNVNPES--QLIQQSEQSESETAGSTKYRLVGVLVHSGQASGGHYYSYIIQRNG
1792 VLAIQLKRFDYDWERECAIKFNDYFEFPRELDMEPYTVAGVAKLEGDDVNPEN--QIIQ-NEQSENEHSGSTKYRLVGVLVHSGQASGGHYYSYIIQRNG

1891 SGGEGERNRWYKFDDGDVTECKMDDDEEMKNQCFGGEYMGEVFDHMMKRMSYRRQKRWWNAYILFYERMDTLDKDSELVKYITELTVSSKPHQVKMPSAI
1895 --GDGERNRWYKFDDGDVTECKMDDDEEMKNQCFGGEYMGEVFDHMMKRMSYRRQKRWWNAYILFYERMDTIDQDDELIRYISELAITTRPHQIIMPSAI
1889 --GDGEKNRWYKFDDGDVTECKMDDDEEMKNQCFGGEYMGEVFDHMMKRMSYRRQKRWWNAYILFYERMDTIDKDSELTKYISELAITTKPHQIKMPSAI

1991 ERSVRKQNVQFMHNRMQYSLEYFQFIRKLLTCNSVYLNSPPGQDHLLPEAEEMAMISIQLAARFLFSTGFHTKKIVRGPASDWYDALCILLRHSKNVRYW
1993 ERSVRKQNVQFMHNRMQYSMEYFQFMKKLLTCNGVYLNPPPGQDHLLPEAEEITMISIQLAARFLFTTGFHTKKVVRGSASDWYDALCILLRHSKNVRFW
1987 ERSVRKQNVQFMHNRMQYSLEYFQFIKKLLTCNSVYLNPPPGQDHLLPEAEEITMISIQLAARFLFTTGFHTKKIVRGPASDWYDALCILLRHSKNVRFW

2091 FAHNVLFAYPNRFSEYLLECPSAEVRGAFSKLIVFIAHFSLQDGPCPTPIASPGPSSQGCDNLSLSEHLFRAVLNLLRREVSEHGRHLQQYFNLFVMYAN
2093 FAHNVLFNVSNRFSEYLLECPSAEVRGAFAKLIVFIAHFSLQDGPCPSPFASPGPSSQAYDNLSLSDHLLRAVLNLLRREVSEHGRHLQQYFNLFVMYAN
2087 FAHNVLFNVSNRFSEYLLECPSAEVRGAFAKLIVFIAHFSLQDGPCPSPFASPGPSSQAYDNLSLSDHLLRAVLNLLRREVSEHGRHLQQYFNLFVMYAN

2191 LGLAEKTQLLKLGVPATFMLVALDEGPGPPIKYQYAELGKLYTVVSQLVRCCDVTSRMQSSINGNPPLANPYGDPNTTTPIMPLQQLVVDILFVRTSYVK
2193 LGVAEKTQLLKLSVPATFMLVSLDEGPGPPIKYQYAELGKLYSVVSQLIRCCNVSSRMQSSINGNPPLPNPFGDPNLSQPIMPIQQNVADILFVRTSYVK
2187 LGVAEKTQLLKLNVPATFMLVALDEGPGPPIKYQYAELGKLYTVVSQLIRCCNVSSRMQSSINGNPPLANPYGDPNLSQPIMALQQNVADILFVRTSYVK

2291 KIIEDCSNSEDTIKLLRFCCWENPQFSSTVLSELLWQVAYSYTYELRPYLDLLLQILFIEDSWQTHRIHNVLKGIPDDRDGLFDTIQRSKNHYQKRAYQC
2293 KIIEDCSNSEETVKLLRFCCWENPQFSSTVLSELLWQVAYSYTYELRPYLDLLLQILLIEDSWQTHRIHNALKGIPDDRDGLFDTIQRSKNHYQKRAYQC
2287 KIIEDCSNSEETIKLLRFCCWENPQFSSTVLSELLWQVAYSYTYELRPYLDLLLQILLIEDSWQTHRIHNALKGIPDDRDGLFDTIQRSKNHYQKRAYQC

2391 IKCMVALFSNCSVAYQILQSNGDLKRKWTWAVEWLGDELERRPYTGNTQYTYNNWSPPVQSNETSNGYFLERSHSARMTLAKACELCPEEEPDEQEALDE
2393 IKCMVALFSNCPVAYQILQGNGDLKRKWTWAVEWLGDELERRPYTGNPQYTYNNWSPPVQSNETSNGYFLERSHSARMTLAKACELCPEEEPDDQDAPDE
2387 IKCMVALFSNCPVAYQILQSNGDLKRKWTWAVEWLGDELERRPYTGNPQYTYNNWSPPIQSNETSNGYFLERSHSARMTLAKACELCPEEEPDDPDAPDE

2491 QDPSPPEDTALYPHSPGSKFQQQNNLPHTQPYTGPAAQHVNNPQRPGPRAQENWEPPEETPQPSQTKD
2493 HESPPPEDAPLYPHSPGSQYQQN-NHVHGQPYTGPAAHHMNNPQRTGQRAQENYEGSEEVSPPQTKDQ
2487 HESSPPEDAPLYPHSPGSQYQQN-NHVHGQPYTGPAAHHMNNPQRTGQRAQENYEGSEEVSPPQTKD-
```

Figure S2. Alignment of Usp9 amino acid sequences. Alignment of zebrafish Usp9 (*Danio rerio*, our sequence), human Usp9 (*Homo sapiens*, XP_005272733.1) and chicken Usp9 (*Gallus gallus*, XP_416773.2). Zebrafish Usp9 bears 90.04% and 92.58% amino acids identity to its counterparts of human and chicken, respectively. Red line indicates the region of ubiquitin-specific protease (USP) domain.
