# Supplementary material for: New Classes of Mind Bomb-Interacting Proteins Identified from Yeast Two-Hybrid Screens
Source: PLoS One. 2014 Apr 8;9(4):e93394. doi: 10.1371/journal.pone.0093394 (PMC3979679; doi:10.1371/journal.pone.0093394)
Supplement: Figure S3 — Alignment of Trabid amino acid sequences. Alignment of zebrafish Trabid (Danio rerio, our sequence), African clawed frog Trabid (Xenopus laevis, NP_001084698), human Trabid (Homo sapiens, CAB64449) and mouse Trabid (Mus musculus, NP_997185). Zebrafish Trabid bears 78.88%, 80.17% and 80.31% amino acids identity to its counterparts of Xenopus, human and mouse, respectively. Blue line indicates the region of Npl4 zinc finger domain (NZF). Green line indicates the region of ankyrin repeat. Red line indicates the region of ovarian tumor protease (OTU) domain. (PDF) [file pone.0093394.s003.pdf]

Figure S3. Alignment of Trabid amino acid sequences

```
1    MTDLGLKWSCEYCTYENWPSAIKCTMCRAQRHNAPIITEEPFKSSSSLDPS---LCTTQGGSTLLICPDSSARPRVRIADELPETSSKWSCHMCTYLNWP  zebrafish
1    MTEDGIKWACEYCTFENWPSAIKCTMCRAPRPSGAIITEEPFKNRTPDVGSM-----EREIGSPLICPDSSARPRVKSSYS-METSSKWSCQICTYLNWP  Xenopus
1    MSERGIKWACEYCTYENWPSAIKCTMCRAQRPSGTIITEDPFKSGSSDVGRDWDPSSTEGGSSPLICPDSSARPRVKSSYS-MENANKWSCHMCTYLNWP  human
1    MSEHGIKWACEYCTYENWPSAIKCTMCRAQRPSGTIITEDPFKSGSSDVGRDWDPSSTEGGSSPLICPDSSARPRVKSSYS-MENANKWSCHMCTYLNWP  mouse

98   RAIRCTQCLSQRQQGSQQHSPLSPSETPQTSGSRPSPVTSDPCEEYNDRNRLNMHAQRWPCSACTYENWPKSLRCVVCDHPKPSGSPETPQQDSEAESAT
95   RAIRCTQCLSQRRTRSPTESPQS-----SGSGLRSIPGPIDPCEEYNDRNKLNIKGQHWTCSACTYENCAKAKKCVVCDHPTPNNMDAIELA----NTDE
100  RAIRCTQCLSQRRTRSPTESPQS-----SGSGSRPVAFSVDPCEEYNDRNKLNTRTQHWTCSVCTYENWAKAKRCVVCDHPRPNNIEAIELA----ETEE
100  RAIRCTQCLSQRRTRSPTESPQS-----SGSGSRPVAFSVDPCEEYNDRNKLNTRTQHWTCSVCTYENWAKAKKCVVCDHPRPNNIEAIELA----ETEE

198  SPSIVNEQERENVRTAGGGGGSRGRLRKLSPPMCKGQAEVKIEL----ASGAVGSDNEQEADFKKLKQIRNRMRRSDWLFLNACAGVVEGDLAAVEAYK
186  ASSIINEQDRARWR-----GGCSSSNSQRRSPPTSKRDS--DMDFQRIELAGAVGSKEEFELDLKKLKQIKNRMRKTDWLFLNACVGVVEGDLSAVEAYK
191  ASSIINEQDRARWR-----GSCSSGNSQRRSPPATKRDSEVKMDFQRIELAGAVGSKEELEVDFKKLKQIKNRMKKTDWLFLNACVGVVEGDLAAIEAYK
191  ASSIINEQDRARWR-----GGCSSGNSQRRSPPTTKRDSEVKMDFQRIELAGAVGTKEELEVDFKKLKQIKNRMKKTDWLFLNACVGVVEGDLAAIEAYK

294  SSGGDIARQLTADEVRILNRPSAFDAGFTLVHLAIRFQRQDMLAVLLTEVSQQTAKCIPALVCPELTEQIRREVAAALHRRKGEFPCYFFTDLVTFTLPA
279  TSGGDIARQLSADEVRLLNRPSAFDVGYTLVHLSIRFQRQDMLAILLTEVAQHAAKCIPAMVCPELTEQIRREIAASVHQRKGDFACYFLTDLVTFTLPA
286  SSGGDIARQLTADEVRLLNRPSAFDVGYTLVHLAIRFQRQDMLAILLTEVSQQAAKCIPAMVCPELTEQIRREIAASLHQRKGDFACYILTDLVTFTLAA
286  SSGGDIARQLTADEVRLLNRPSAFDVGYTLVHLAIRFQRQDMLAILLTEVSQQAAKCIPAMVCPELTEQIRREIAASLHQRKGDFACYFLTDLVTFTLPA

394  DIEDLPPNVQEKLFDEVLDRDVQKELEEESPIINWSLELGTRLDSRLYALWNRTAGDCLLDSVLQATWGIYDKDSVLRKSLNDSLHDCSHWFYTRWKEWE
379  DIEDLPPTVQEKLFDEVLDRDVQKELEEESPIINWSLELGTRLDSRLYALWNRTAGDCLLDSVLQATWGIYDKDSVLRKALHDSLRDCSHWFYSRWKEWE
386  DIEDLPPTVQEKLFDEVLDRDVQKELEEESPIINWSLELATRLDSRLYALWNRTAGDCLLDSVLQATWGIYDKDSVLRKALHDSLHDCSHWFYTRWKDWE
386  DIEDLPPTVQEKLFDEVLDRDVQKELEEESPIINWSLELATRLDSRLYALWNRTAGDCLLDSVLQATWGIYDKDSVLRKALHDSLHDCSHWFYTRWKDWE

494  SWYSQSFGLHFSLREEQWQEDWAFILSLASQPGASLEQTHVFVLAHILRRPIIVYGVKYYKSFRGETLGYTRFQGVYLPLLWEQSFCWKSPIALGYTRGH
479  SWYSQSFGLHFSLREEQWQEDWAFILSLASQPGASLEQTHIFVLAHILRRPIIVYGVKYYKSFRGETLGYTRFQGVYLPLLWEQSFCWKSPIALGYTRGH
486  SWYSQSFGLHFSLREEQWQEDWAFILSLASQPGASLEQTHIFVLAHILRRPIIVYGVKYYKSFRGETLGYTRFQGVYLPLLWEQSFCWKSPIALGYTRGH
486  SWYSQSFGLHFSLREEQWQEDWAFILSLASQPGASLEQTHIFVLAHILRRPIIVYGVKYYKSFRGETLGYTRFQGVYLPLLWEQSFCWKSPIALGYTRGH

594  FSALVAMENDGYDNRGAGANLNTDDDVTVTFLPLVDSERKLLHIHFLSAQEMGTEEQQERMLRQWMDCCVTEGGVLVAMQKSSRRRNHPLVTQMVEKWLD
579  FSALVAMENDGFDNRGAGANLNTDDDVTVTFLPLVDSERKLLHVHFLSAQELGNEDQQEKLLREWMDCCVTEGGVLVAMQKSSRRRNHPLVTQMVEKWLD
586  FSALVAMENDGYGNRGAGANLNTDDDVTITFLPLVDSERKLLHVHFLSAQELGNEEQQEKLLREWLDCCVTEGGVLVAMQKSSRRRNHPLVTQMVEKWLD
586  FSALVAMENDGYGNRGAGANLNTDDDVTITFLPLVDSERKLLHVHFLSAQELGNEEQQEKLLREWLDCCVTEGGVLVAMQKSSRRRNHPLVTQMVEKWLD

694  GYRQLAACPTLSDGEEEEEDEDE
679  GYRQIRPCTALSDGEEDEDDEDE
686  RYRQIRPCTSLSDGEEDEDDEDE
686  RYRQIRPCTSLSDGEEDEDDEDE
```

Figure S3. Alignment of Trabid amino acid sequences. Alignment of zebrafish Trabid (*Danio rerio*, our sequence), African clawed frog Trabid (*Xenopus laevis*, NP_001084698), human Trabid (*Homo sapiens*, CAB64449) and mouse Trabid (*Mus musculus*, NP_997185). Zebrafish Trabid bears 78.88%, 80.17% and 80.31% amino acids identity to its counterparts of *Xenopus*, human and mouse, respectively. Blue line indicates the region of Npl4 zinc finger domain (NZF). Green line indicates the region of ankyrin repeat. Red line indicates the region of ovarian tumor protease (OTU) domain.
