# Supplementary material for: New Classes of Mind Bomb-Interacting Proteins Identified from Yeast Two-Hybrid Screens
Source: PLoS One. 2014 Apr 8;9(4):e93394. doi: 10.1371/journal.pone.0093394 (PMC3979679; doi:10.1371/journal.pone.0093394)
Supplement: Figure S4 — Alignment of Fih-1 amino acid sequences. Alignment of zebrafish Fih-1 (Danio rerio, our sequence), human Fih-1 (Homo sapiens, NP_060372.2) and mouse Fih-1 (Mus musculus, NP_795932.2). Zebrafish Fih-1 bears 81.76% and 82.35% amino acids identity to its counterparts of human and mouse, respectively. Purple line indicates the region of Jmjc domain. (PDF) [file pone.0093394.s004.pdf]

Figure S4. Alignment of Fih-1 amino acid sequences

```
1    MAE---------TDGAAAFTELRDPDWDESQLRQYTFPTRQIPRLSHTDPRAEVLINNEEPVVLTDTSLVYPALKWDIPYLQENIGNGDFSVYI ENHKF   zebrafish
1    MAATAAEAVASGSGEPREEAGALGPAWDESQLRSYSFPTRPIPRLSQSDPRAEELIENEEPVVLTDTNLVYPALKWDLEYLQENIGNGDFSVYSASTHKF   human
1    MAATAAEVAASGSGEAREEAEAPGPAWDESQLRSYSFPTRPIPRLSQSDPRAEELIENEEPVVLTDTNLVYPALKWDLEYLQENIGNGDFSVYSASTHKF   mouse

92   LYYDEKKMVNFQD V K RRIEMKFSEFVDKMHQTEEQGGKGRVYLQQTLNDTVGRKIVVDFLGFNWNWINKQQAKRNWGPLTSNLLLIGMEGNVTPAHY
101  LYYDEKKMANFQNFKPRSNREEMKFHEFVEKLQDIQQRGGEERLYLQQTLNDTVGRKIVMDFLGFNWNWINKQQGKRGWGQLTSNLLLIGMEGNVTPAHY
101  LYYDEKKMGNFQNFKPRSNREEIKFHEFVEKLQAIQQRGGEERLYLQQTLNDTVGRKIVMDFLGFNWNWINKQQGKRGWGQLTSNLLLIGMEGNVTPAHY

192  DEQQNFFAQIKGHKRCILFPPDQFDCLYPYPVHHPCDRQSQVDFENPDYDKFPNFKNAVGYEAVVGPGDVLYIPMYWWHHIESLLNGGETITVNFWYKGA
200  DEQQNFFAQIKGYKRCILFPPDQFECLYPYPVHHPCDRQSQVDFDNPDYERFPNFQNVVGYETVVGPGDVLYIPMYWWHHIESLLNGGITITVNFWYKGA
200  DEQQNFFAQIKGHKRCILFPPDQFECLYPYPVHHPCDRQSQVDFDNPDYERFPNFRNVVGYETVVGPGDVLYIPMYWWHHIESLLNGGITITVNFWYKGA

292  PTPKRIEYPLKAHQKVAIMRNIEKMLGEALGDPHEVGPLLNMMIKGRYDHGLS
300  PTPKRIEYPLKAHQKVAIMRNIEKMLGEALGNPQEVGPLLNTMIKGRYN----
300  PTPKRIEYPLKAHQKVAIMRNIEKMLGEALGNPQEVGPLLNTMIKGRYN----
```

Figure S4. Alignment of Fih-1 amino acid sequences. Alignment of zebrafish Fih-1 (*Danio rerio*, our sequence), human Fih-1 (*Homo sapiens*, NP_060372.2) and mouse Fih-1 (*Mus musculus*, NP_795932.2). Zebrafish Fih-1 bears 81.76% and 82.35% amino acids identity to its counterparts of human and mouse, respectively. Purple line indicates the region of Jmjc domain.
